# Social and Psychiatric Aspects of the Emigration Problem in Ukraine

**DOI:** 10.25122/jml-2019-0122

**Published:** 2020

**Authors:** Olena Petrivna Venger, Yuriy Ihorovych Mysula, Marianna Serhiivna Mysula

**Affiliations:** Department of Psychiatry, Narcology and Medical Psychology, I. Horbachevsky Ternopil State Medical University, Ternopil, Ukraine

**Keywords:** Emigrants, depressive disorders, socio-psychological, clinical-psychopathological aspects, treatment

## Abstract

The article presents data on a new scheme of complex treatment and rehabilitation of emigrants with psychogenic, endogenous and organic depressive disorders, as well as the results of comparative analysis of the effectiveness of this scheme, compared to the traditional one.

The use of the proposed treatment scheme also allowed to reduce the severity of anxiety, indicators of the severity of psychopathological symptoms, improve the quality of life among emigrants suffering from depressive disorders of different genesis.

The received results allow us to recommend the scheme we have developed for implementation in the complex treatment of emigrants suffering from depressive disorders of psychogenic, endogenous, and organic genesis.

## Introduction

The problem of emigration in Ukraine is one of the most pressing state and social problems. Emigration is one of the most massive social processes in the world, which has shown a pronounced tendency to increase in recent years. Based on the information from Ukrainian embassies abroad, the Ministry of Foreign Affairs reports that up to 2.5 million citizens are employed outside the country. The data of the Ministry of Social Policy of Ukraine obtained based on the analysis of the labor market, made it possible to determine the volume of labor migration, which is approximately 3 million persons [1]. According to the World Bank, Ukraine ranks fifth globally by the number of migrants; migration between Ukraine and the Russian Federation has the second or third place in the world by the amount of human traffic [2, 3]. At the same time, official emigration represents a small part of the total emigration; the majority of migrants (from 60 to 90%) move abroad with the declared aim of tourism, visiting relatives, and others with subsequent employment (legal or illegal) in recipient countries [4]. According to the State Statistics Committee, 35.1% of the migrants had a residence permit and work permit, 39.3% had temporary registration, and 25.6% of them did not hold an official status to stay abroad. According to the International Organization for Migration, in 2015, the proportion of families whose members were currently working abroad was 5.5%. Simultaneously, the number of persons unofficially working increased significantly from 20.6% in 2008 to 40.5% in 2015 [5].

In recent years, due to Russian aggression towards the East of Ukraine, there has been a tendency for a rapid increase in the number of Ukrainian migrants. If the intention to emigrate was present in 12.3% of the respondents in 2005, the percentage was already 65.0% in 2015 [6]. In 2015, European countries registered more asylum applications than in the last decade, and the absolute number of appeals per year is the highest over the past 15 years, the largest number of Ukrainians moving to Poland, the Czech Republic, Germany, and Italy. The European Union (EU) government structures expect that these migrants will stay in recipient countries for several years [7].

Migration is seen as an important factor in influencing a person’s mental health. Some authors believe that emigration provokes manifestation or exacerbation of endogenous mental illness. At the same time, the issues related to the peculiarities of the depressive disorders in emigrants have not been studied sufficiently. In particular, the manifestations of depression concerning the emigration factor have not been investigated, which significantly complicates the development of targeted therapeutic approaches to the treatment and prevention of depressive disorders in this population category. This study was based on the research and analysis of clinical-psychopathological phenomenology of depression to develop the system of measures for treating depressive disorders in emigrants.

## Literature Review

Migration is accompanied by the transformation of the whole system of socio-psychological relationships, including interpersonal, family, parental, labor, and leads to significant tension of psychological mechanisms, which finally can lead to depressive and anxiety disorders, somatization, and post-traumatic stress disorder. The process of adaptation of migrants in a recipient country can be challenging and require a fundamental psychological and sociocultural restructuring, defined by the term “stress of acculturation” [8, 9].

N.V. Kondrashova believes that the interdisciplinary nature of the migration problem necessitates the use of a set of research methods based on the implementation of ethno-sociological and cultural approaches, which ensures obtaining the results that objectively reflect the current state of the problem, makes it possible to predict the development of this process and to give scientifically substantiated recommendations [10].

According to N.S. Palagina, the problem of adaptation of forced migrants has a complex interdisciplinary nature, while the socio-psychological aspect of the adaptation of migrants is not enough investigated. In the process of social and psychological adaptation of migrants, there are significant changes in the personal plan manifested in decreased self-esteem and level of aspirations, deformation of value orientations, and social attitudes. The author highlights that most of the forced migrants are non-adapted, which is manifested in the hypothymia type of response, the chaotic nature of the activity, in trend to take a protective position, externality and escapism, a decreased emotional state, emotional tension, and a tendency to groundless concern about minor issues, irritability, and anxiety. The situation of forced migration can be characterized as a crisis and even extreme, the one that poses a threat to health and life. At the same time, the success of the socio-psychological adaptation of migrants is determined by psychological and non-psychological conditions, in particular, socio-demographic affiliation, personal characteristics, life experience, degree of similarity, and differences between cultures, the communicative potential of the subject, creativity, and others. In the process of socio-psychological adaptation of migrants, there are clearly revealed phases (stages) that characterize the dynamics of adaptation processes and the success of adaptation in general. A comprehensive program of assistance, which includes a system of social, psychological, and educational measures, can optimize socio-psychological adaptation by main indicators, such as adaptation to the sociocultural environment, emotional stability, social frustration, and mental state [11].

L.A. Shaygerova emphasizes that the complex process of adaptation in the situation of migration leads to changes in the identity of the migrant’s personality that reach a crisis level. The crisis of identity among migrants manifests itself in the different profound transformations of its contents, occurring on the personal and group levels. So, the overcoming of the identity crisis is an essential condition for the successful adaptation of the individual in the situation of forced migration. However, it often reaches such depth and strength that personal resources are not enough and the person requires psychological help for overcoming it. Such help is the correction of violations of the motivational and necessity sphere, restoring of positive identity, integrity, and integration, development of constructive strategies for overcoming, building up and integrating into the content of the personality’s identity of the values and elements of the host culture [12].

It is evident that the nature of emigration – forced or voluntary – is extremely important for the negative impact on the psyche of an individual. R. Rice and D. Bhurra et al. emphasize that the problem of forced migration, which has a powerful negative impact on the state of migrants’ mental health, has recently become a result of political instability, poverty, terrorism, and other negative social factors [13]. From the very beginning, the readiness for self- development, and self-realization of a voluntary migrant is quite high, and the situation of emigration itself is an opportunity to realize this readiness. In the case of forced migration, when the reason for migration lies outside the individual and is completely dictated by external circumstances, which greatly reduces his readiness to adapt in the changing circumstances, is often accompanied by severe psychological trauma [14, 15]. At the same time, M.A. Maximov emphasizes that any migration is essentially forced, as it is caused by the inability of the individual to continue to live in the homeland conditions.

Z.H. Lepshokova notes that the characteristics of the ethnic identity of the migrants are related to certain acculturation strategies: the expressiveness of the national identity of the migrants is positively related to the separation strategy, the expressiveness of the civic identity – with the strategy of assimilation. At the same time, the integration strategy is positively associated with all components of the psychological well-being of the individual and with a high level of sociocultural adaptation of migrants. Assimilation, separation, and marginalization strategies have a predominantly negative relationship with the components of mental well-being and mental health (anxiety and depression) [16].

Emigration is considered as a factor provoking a manifestation or exacerbation of endogenous mental illness [17-19]. Emotional instability, anxiety, social introversion, conformance on the behavioral level, suspicion, weak integration of personality traits in the form of emotionality and frustration, low level of frustration tolerance are revealed in the structure of the personality of the emigrants. The structure of mental disorders of emigrants is dominated by psycho-dysadaptation, post-traumatic stress disorder, neurasthenia, prolonged depressive reaction and moderate depressive episodes [20-23].

Numerous studies have found that the situation of migration determines the high dissatisfaction of the individual with life changes and leads to the development of depression, psychosis, paranoid and schizoid disorders.

C.A. Zaiontz et al. note that migration is a factor that provokes, exacerbates and participates in the formation of a psychopathological picture of some severe mental disorders, especially affective disorders; the authors consider it necessary to study the level of sociocultural integration and psychological support of migrants in recipient countries [24].

B.L. Zhong et al. report significant psychoemotional stress experienced by migrants, especially difficulties of adaptation in the new social environment, intense work, farness from the family, and financial difficulties [25].

R.A. Yaskevich et al. emphasize the peculiarities of older migrants’ adaptation severity; in addition to the exacerbation of somatic diseases, in the period of adaptation, depressive symptoms can be seen in most individuals. The women have depressive symptoms 1.8 times more often and anxiety disorders 3.2 times more often than men. The authors recommend the use of a special rehabilitation psychotherapeutic program for such migrants [26].

M. Shen et al. note that the presence of depressive and anxiety disorders in migrant parents affects the normal functioning of the whole family and causes behavioral disorders in children [27].

N.S. Mahajan et al. emphasize that emigration is a risk factor for psychosis. The most common diseases are mood disorders - women have a greater prevalence of a major depressive episode with melancholic symptoms, while manic episodes are more frequent in men. In this case, mental disorders are especially prevalent among the second generation of emigrants, which is associated with the effects of migration stress, discrimination, and acculturation in the early years of life [28].

B.L. Zhong et al. report a greater risk of a major depressive disorder during the life of labor migrants [29]. Similar data is given by C.K. Hoia et al.; in their study, correlations of the severity of depressive manifestations with characteristics of social contacts and emotional support were shown. The authors emphasize that interventions that promote the adaptation of migrants are an effective way of reducing the symptoms of depression [30].

M.E. Beutel et al. point out that, despite the actuality of the problem of emigration in Germany, the studies of the migrant’s mental health are extremely insufficient. Based on large-scale research (almost 15,000 migrants), the authors found a high prevalence of depressive disorders, anxiety disorders, and suicide among first-generation migrants, while the mental health of the second generation of migrants did not differ from ethnic Germans; their socio-economic status was also high [31].

M. Schouler-Ocak indicates that migrants suffer from specific psychiatric disorders (mood disorders, somatoform disorders, post-traumatic stress disorder), and the prevalence of mental disorders in this population is greatly increased [32].

D. Bhugra et al. showed that migrants are more prone to mental illness, a fact that should be taken into account in public health and social policy programs.

The mental health of migrants has become a key problem in current healthcare issues in EU member states. H.J. Salize et al. indicate that mental health services in the recipient countries are not able to provide adequate psychiatric care to migrants because of a growing stream of mental illnesses, limited health resources, the reduced number of trained professionals, and new legal requirements. Several studies substantiate the need to involve a variety of state and community structures that provide language, cultural and social adaptation of migrants with free access to mental health services in the system of psychiatric help.

The experience, gained by migrants in combat zones, crisis regions, and during escapes, increases the risk of primary and secondary traumatism with further psychiatric disorders. In addition to post-traumatic stress disorder, major depressive disorders, anxiety disorders, and psychosis are more likely to occur in migrants than in the general population. Recent studies have shown that around half of forced migrants suffer from mental illnesses such as depression, post-traumatic stress disorder, and anxiety disorders. In Germany, about 5 thousand sessions of psychotherapy are held annually, which is only 4% of the need.

Treatment and rehabilitation approaches for depressive disorders of migrants are currently in the stage of active formation. Understanding the importance of correction of depressive symptoms among migrants has motivated scientists from different countries to find new methods of treatment and prevention of depressive disorders in this category of patients.

Looking at the life-threatening clinical manifestations, complications and the presence of high suicidal danger, it is clear to see that severe depression belongs to the category of urgent pathologies. The leading deontological requirement for the provision of medical care to patients with depression is the most rapid possible achievement of the therapeutic effect with the appointment of individually adequate intensive care. It requires a comprehensive multivariate assessment of the patient’s condition, including the operative diagnosis of depression and comorbid states, clinical- psychopathological and syndromal evaluation of depression, characteristics of its course, comorbidity and pathogenesis. The authors highlight the need for complex treatment using different medication groups, non-drug therapy with subsequent supportive treatment for reducing the symptoms of the current episode and preventing exacerbations, as well as a prophylactic treatment to avoid relapses.

It is evident that migrants need special therapeutic approaches to the treatment of depressive disorders, taking into account the socio-psychological characteristics of this contingent of patients. However, analyzing the modern scientific literature on pharmacological therapy of depressive disorders in emigrants (remigrants), it should be noted that this problem remains practically not resolved. Somewhat more studied are measures for psychosocial therapy of affective disorders in emigrants and remigrants.

Specialists of the European Psychiatric Association have formulated recommendations for reducing the risk of migrants’ mental disorders.

In the public sphere, a clear policy that takes into account employment, provision of housing, the satisfaction of the physical and intellectual needs of migrants, with appropriate resources that has to be freely available to migrants, provision of the system of social-psychological support of migrants by skilled personnel, adequate information policy, collection and analysis of the necessary information have been implemented.

In the field of mental health, the availability to physicians for necessary information, cultural awareness, and training should become an obligatory part of professional competence; it should be the part of training plans for medical staff that provide psychiatric and psychological help to migrants and the range of medical (psychiatric) services should be sufficient and cover all main areas, being provided with all necessary resources. Much attention is paid to the importance of psycho-educational activities, which include giving practical advice to migrants all the necessary knowledge about illnesses. Such information should be problem-oriented.

The psychological assistance to emigrants for effective psychological adaptation is determined by the following directions: changes in the living conditions of the emigrant according to his educational and professional level, the change in the hierarchy of needs and in the system of meaningful goals, as well as the increase of intrapsychic adaptation in the process of psychological correction.

Cognitive-behavioral therapy (CBT) is one of the most promising psychotherapeutic directions in the treatment of depressive disorders. CBT refers to a group of interventions that are among the best-known empirically-supported treatments for depression. CBT is based on the premise that inaccurate beliefs and maladaptive information processing (forming the bases for repetitive negative thinking) have a causal role in the etiology and maintenance of depression. This ‘cognitive model’ posits that when maladaptive thinking is corrected, both acute distress and the risk for subsequent symptom return will be reduced [33]. At the same time, D. Bhugra et al. notes that, in some cases, depressive disorders in migrants do not require medical intervention and can be localized by the religious approaches [34].

Thus, the analysis of modern scientific literature devoted to the problem of depressive disorders in emigrants and remigrants shows that its development is not enough. The contradictory nature of the scientific data of the influence of factors of emigration and remigration on the emergence and the course of depressive disorders, the lack of programs of complex pharmacological and psychosocial correction, causes the relevance of further research of the clinical, psychopathological and psychiatric aspects of this problem, as well as the development of modern approaches to integrated treatment and rehabilitation of emigrants and remigrants with depressive disorders.

Based on this research, the scheme of integrated treatment and rehabilitation of emigrants with psychogenic, endogenous, and organic depressive disorders was developed and implemented in the health care practice. A comparative analysis of the effectiveness of this scheme and the traditional one was conducted.

The proposed treatment and rehabilitation schemes are based on the principles of integrated, individualized, and differentiated approaches to ensuring the phases and continuity of treatment and rehabilitation measures. Its goals are to stop, as quickly as possible, the manifestations of depressive disorders (depressive, anxiety-depressive, asthenic-depressive, apathetic depression) and ensure maximal restoration of the ability to work, social functioning of the patient, prevention of relapses of depressive disorders, providing early socialization and social adaptation, taking into account specific features identified at emigrants and reemmigrants, with the involvement of family and public organizations. Targets of therapy are clinical manifestations of depressive disorders (low mood, anhedonia, increased fatigue, anxiety, agitation), social maladaptation and disorders of microsocial communications, pathological behavior patterns associated with the presence of depressive disorders, as well as factors of emigration and remigration.

A comparative assessment of the effectiveness of the proposed treatment and rehabilitation scheme relative to the traditional one was done by comparing clinical and psychological and psychometric data, as well as indicators of quality of life.

The proposed scheme is implemented in four stages related to the continuity of treatment and diagnostic measures.

• The first stage – diagnosis. It includes clinical and psychopathological evaluation of the existing psycho-emotional disorders in the patient, analysis of anamnestic data, clinical symptoms, dynamics and prognosis of the disease, the relationship of clinical and socio-psychological factors.
• The second stage – complex treatment. Includes a complex of biological therapy and psychotherapy aimed at reducing depression, normalization of the psycho-emotional state, social adaptation and rehabilitation. Differentiated psychotherapeutic correction of emigrants includes the use of rational psychotherapy, cognitive-behavioral therapy and group psychotherapy. In emigrants with psychogenic depressive disorders, psycho-educational therapy could also be used. The criterion of the effectiveness of therapy is a stable (at least two weeks) normalization of the psycho-emotional state, the absence of clinical signs of depressive disorder, the development of an adequate emotional response to real-life circumstances, including emigration, giving up destructive patterns and developing constructive patterns of behavior.
• The third rehabilitation stage is aimed at the formation of a stable adequate emotional and behavioral pattern, maximum adaptation and rehabilitation of the patient, prevention of relapses of the depressive disorder. The drug therapy is similar to the one used in the treatment stage, with the correction of doses of medications, depending on the current state of the patient. Differentiated psychotherapeutic work includes the use of cognitive-behavioral psychotherapy in emigrants and a combination of cognitive-behavioral therapy, family psychotherapy and autogenous training in remigrants. At this stage, there are also measures for social rehabilitation and the readaptation of the patient.
• The fourth stage - prophylactic - is aimed at maintaining the normal psycho-emotional state, effective resistance to stress, and prevention of relapse of depressive disorder. Medication therapy includes the treatment of the main disease in depressive disorders of the organic genesis and the seasonal prevention of endogenous depressions. Psychotherapy, the use of techniques of self- regulation, and measures for social adaptation could be used as well.

## Conclusions

In groups of emigrants that suffer from depressive disorders, the proposed scheme of dynamics therapy showed a higher efficiency, compared with the traditional scheme in terms of recovery and improvement of the general condition, reduction of psychopathological symptoms, and increased quality of life.

The obtained results allow us to recommend the scheme we have developed for implementation in the complex treatment of emigrants suffering from depressive disorders of psychogenic, endogenous and organic genesis.

## Conflict of Interest

The authors declare that there is no conflict of interest.
